# The Effects of Capsaicin on Gastrointestinal Cancers

**DOI:** 10.3390/molecules26010094

**Published:** 2020-12-28

**Authors:** George Denis Alexandru Popescu, Cristian Scheau, Ioana Anca Badarau, Mihai-Daniel Dumitrache, Ana Caruntu, Andreea-Elena Scheau, Daniel Octavian Costache, Raluca Simona Costache, Carolina Constantin, Monica Neagu, Constantin Caruntu

**Affiliations:** 1 Department of Medical Oncology II, “Prof. Dr. Alexandru Trestioreanu” Institute of Oncology, 022328 Bucharest, Romania; george-denis-alexandru.popescu@rez.umfcd.ro; 2 Department of Physiology, “Carol Davila” University of Medicine and Pharmacy, 050474 Bucharest, Romania; anca.badarau@umfcd.ro (I.A.B.); costin.caruntu@gmail.com (C.C.); 3 Departament of Pneumology IV, “Marius Nasta” Institute of Pneumophtysiology, 050159 Bucharest, Romania; dumitrache_mihai_daniel@yahoo.com; 4 Department of Oral and Maxillofacial Surgery, “Carol Davila” Central Military Emergency Hospital, 010825 Bucharest, Romania; ana.caruntu@gmail.com; 5 Department of Preclinical Sciences, Faculty of Medicine, “Titu Maiorescu” University, 031593 Bucharest, Romania; 6 Department of Radiology and Medical Imaging, Fundeni Clinical Institute, 022328 Bucharest, Romania; andreea.ghergus@gmail.com; 7 Department of Dermatology, “Carol Davila” Central Military Emergency Hospital, 010825 Bucharest, Romania; daniel_costache@yahoo.com; 8 Gastroenterology and Internal Medicine Clinic, “Carol Davila” Central Military Emergency Hospital, “Carol Davila” University of Medicine and Pharmacy, 050474 Bucharest, Romania; raluca.costache@umfcd.ro; 9 Immunology Department, Victor Babes National Institute of Pathology, 050096 Bucharest, Romania; caroconstantin@gmail.com (C.C.); neagu.monica@gmail.com (M.N.); 10 Department of Pathology, Colentina University Hospital, 020125 Bucharest, Romania; 11 Faculty of Biology, University of Bucharest, 76201 Bucharest, Romania; 12 Department of Dermatology, Prof. N.C. Paulescu National Institute of Diabetes, Nutrition and Metabolic Diseases, 011233 Bucharest, Romania

**Keywords:** capsaicin, digestive cancer, tumorigenesis, apoptosis, molecular signaling

## Abstract

Gastrointestinal (GI) cancers are a group of diseases with very high positions in the ranking of cancer incidence and mortality. While they show common features regarding the molecular mechanisms involved in cancer development, organ-specific pathophysiological processes may trigger distinct signaling pathways and intricate interactions with inflammatory cells from the tumoral milieu and mediators involved in tumorigenesis. The treatment of GI cancers is a topic of increasing interest due to the severity of these diseases, their impact on the patients’ survivability and quality of life, and the burden they set on the healthcare system. As the efficiency of existing drugs is hindered by chemoresistance and adverse reactions when administered in high doses, new therapies are sought, and emerging drugs, formulations, and substance synergies are the focus of a growing number of studies. A class of chemicals with great potential through anti-inflammatory, anti-oxidant, and anti-tumoral effects is phytochemicals, and capsaicin in particular is the subject of intensive research looking to validate its position in complementing cancer treatment. Our paper thoroughly reviews the available scientific evidence concerning the effects of capsaicin on major GI cancers and its interactions with the molecular pathways involved in the course of these diseases.

## 1. Introduction

Gastrointestinal cancer is a broad term encompassing the malignant tumors affecting the digestive tract and annex glands, some of them being among the most aggressive and resilient cancers in humans and causing 4.5 million global deaths every year [1]. A large part of current research is dedicated to studying the pathogenesis and evolution of gastrointestinal cancers in an effort to obtain earlier diagnoses and to improve the management and treatment of these diseases. The major scientific investment in this field continues to uncover new molecular mechanisms governing the dissemination of cancer cells while new surgical methods are developed in the attempt to minimize tissue trauma and improve patient recovery times [2]. However, pertaining to the pharmaceutical treatment of GI cancers, the existing drugs have less than ideal performances due to incomplete disease control and the onset of adverse effects and chemoresistance. Emerging studies are proposing immune checkpoint inhibitors, DNA-targeting agents, antiangiogenic drugs, and newly-developed immunotherapeutic substances for a variety of GI cancers, yet the validation of these therapies for clinical use is a long and arduous process [3,4].

Lately, phytochemicals are viewed with increasing interest due to their demonstrated anti-inflammatory and anti-oxidant effects and are considered for use as antitumoral agents based on the rising number of studies where they demonstrated efficiency against various cancers [5,6]. In this regard, capsaicin is one of the most promising substances as it has shown anticancer activity by diminishing the tumoral progression, decreasing the metastasis rates, and increasing survival in a variety of studies on different cancers [7,8].

## 2. Capsaicin in Gastrointestinal Cancer

Capsaicin (trans-8-methyl-N-vanillyl-6-nonenamide) is the major component that produces the burning sensation when ingesting hot peppers. Capsaicin is an alkaloid found in various species of hot peppers and is part of the Capsaicinoids family, which consists mainly of capsaicin, dihydrocapsaicin, nordihydrocapsaicin, homodihydrocapsaicin, and homocapsaicin.

Capsaicin agonists consist of three molecular regions: a vanillyl group named “head” with the structure of an aromatic ring, an amide bond (“neck”), and a hydrophobic side-chain referred to as aliphatic “tail”; structural variety in these regions may influence the agonist activity potency, and this is particularly important for synthetic derivatives, as new studies seek out artificial compounds with superior potency and less or no pungency [9]. While capsaicin accounts for more than 80% of capsaicinoids, its analogues are very similar in structure and show variations mainly in the length and degree of double bonds presence in the “tail” [10].

Capsaicin is well absorbed in the gastrointestinal tract when administered orally, but also through the skin when administered topically because of its properties: hydrophobic aggregate, presenting a nonpolar phenolic structure. In oral administration, absorption of capsaicin in the gastrointestinal tract is a passive process with up to 90% rates of absorption [11]. A study on the gastrointestinal absorption of capsaicin in rats has shown that the main site of absorption is the jejunum, followed by the ileum and stomach [12]. However, capsaicin is almost entirely metabolized in the liver with the production of several metabolites: 16- and 17-hydroxycapsaicin, 16,17-dehydrocapsaicin, 5,5′-dicapsaicin, vanillylamine, and vanillin [13,14]. There are reports that these metabolites may bind to TRPV1, albeit with less potency than capsaicin, and may exhibit similar yet weaker effects compared to capsaicin. However, conclusive data related to gastrointestinal cancers are scarce [15,16,17]. Capsaicinoids escaping liver metabolization may be found unchanged in the urine, albeit in small concentration compared to the orally administered dose [12,18]. However, the intake of dietary capsaicin varies significantly between individuals and populations and may average anywhere between several milligrams to 10 or more grams per day [19,20].

The capsaicin receptor is a transient cation channel type belonging to the subfamily V member 1 of TRP receptor group (TRPV1) [21,22], a channel also activated by heat [23]. This is how capsaicin produces the burning sensation [24]. The docking to TRPV1 is performed in a specific orientation, with the “tail” pointing upwards and allowing a more flexible configuration while the “head” is disposed deep into the binding pocket causing activation through structural changes [25,26]. TRPV1 is expressed in the digestive tract, but with varied distribution depending on the presence of inflammation or tumors, which can also induce the expression of calcitonin gene-related peptide (CGRP) or substance P (SP) [27].

Capsaicin targets some signaling pathways involved in carcinogenesis and tumor invasion, but its global impact is not entirely known, despite the observation that it may disturb cancer cell metabolism [28] implying some anti-neoplastic biologic effects. However, in most cases, this action on cancer cells’ metabolism seems to be independent of TRPV1 [29].

In numerous in vivo studies, capsaicin has demonstrated anticancer effects by diminishing tumor cells progression in mice. The antitumor properties of capsaicin have been explored in a series of other studies as well [30,31]. In the gastrointestinal tract, capsaicin shows antitumoral effects in gastric cancer [32,33], cholangiocarcinoma [34], hepatocellular carcinoma [35], pancreatic cancer [36], and colon cancer [37,38]. A study from 1998 has established that capsaicin caused programmed cell death by increasing the ROS (reactive oxygen species) production, inducing cell apoptosis by affecting a series of cellular signaling networks such as tumor suppressor p53 pathway [39]. Various in vitro and in vivo models have been used to demonstrate the antitumoral effects of capsaicin (Table 1).

However, carcinogenic effects of capsaicin on gastrointestinal tumors were also cited in multiple studies [55]. Nevertheless, these reports are scarce and often the findings have not been confirmed by newer studies and hence published papers. Most of these reports are based on animal models that use induction of cancer through cancer cell lines that undergo adaptations and show particular signaling and genomics. Extrapolation to human subjects is in most cases impossible due to the large number of confounding variables and specific differences [56]. However, since all these observations might also be applied when considering the anti-tumor effects, a summary of some of the most important reports on carcinogenic effects of capsaicin on different study models is depicted in Table 2.

### 2.1. Esophageal Squamous Cell Carcinoma

Esophageal cancer is an extremely aggressive cancer due to its poor survival rate and ranks sixth in cancer mortality [62]. Some of the most common risk factors in developing esophageal cancer are smoking, alcohol, diet (hot tea, high red meat intake, and low vegetable intake), genetics (Tylosis-an autosomal dominant disease), obesity, low socioeconomic status, and caustic injury [63]. A recent study has demonstrated that capsaicin has the power to inhibit tumor glycolysis in esophageal squamous cell carcinoma (ESCC) by acting on hexokinase-2 expression, an enzyme participating in the process of glycolysis, a mechanism that is involved in the rapid growth of cancer cells [40]. In this study, it was revealed that capsaicin downregulates the hexokinase-2 expression and inhibits cancer cells glycolysis. In vitro results showed that capsaicin induces a suppressive effect on esophageal squamous cell carcinoma cells and the cellular response is dose-dependent [40]. The activity of hexokinase-2 was investigated in other malignancies such as non-small cell lung cancer and breast cancer and it was suggested that hexokinase-2 has a negative impact on the treatment of cancer by promoting chemoresistance and the inhibition of glycolysis through hexokinase-2 increases the cancer cells chemotherapy sensitivity [64,65,66].

In another recent in vitro study, capsaicin inhibited the dissemination of ESCC cells by activating the 5’ AMP-activated protein kinase (AMPK) signaling pathway that caused a decrease in the expression of matrix metalloproteinase (MMP) 9, a known regulator of cancer invasion and migration [41].

Conversely, in a 2019 study, Huang et al. presented salient findings regarding the overactivation of TRPV1 by capsaicin and TRPV4 stimulation by hypoosmotic solutions. In an in vitro study on Eca109 and TE-1 ESCC cell lines, the vanilloid receptors were functionally expressed in the tumor cells and their overactivation promoted tumor growth and invasion. Capsaicin exposure between 1 and 5 days stimulated the proliferation of ESCC cells at a concentration of 15 µM, which is below the EC50 for the induction of [Ca^2+^]_i_ increase, while the apoptotic effects on cancer cells were obtained at concentrations above the EC50 threshold [57]. Further in vivo studies are needed for validating these findings and advancing the understanding of the capsaicin involvement in the networked pathways regulating tumor development and dissemination.

### 2.2. Gastric Cancer

Gastric cancer is one of the leading causes of death by cancer worldwide despite a sustained decrease in incidence due to continuous efforts in restricting the exposure to various risk factors [67].

#### 2.2.1. Antitumoral Effects of Capsaicin in Gastric Cancer

Various studies have suggested that capsaicin could act as an important agent against gastric cancer. Several in vitro studies have shown that capsaicin has the potential to inhibit the proliferation of gastric cancer cells and to promote their apoptosis. It was revealed that these actions are performed by reducing the expression of Bcl-2 [68,69]. Similar results were emphasized in an in vitro study on a human gastric adenocarcinoma cell line which has shown that exposure to a dose of 10 to 200 μmol/L of capsaicin reduces the BCL-2 expression in these cells [32]. BCL-2 is an antiapoptotic protein, responsible for the regulation of the transmembrane calcium fluxes. Dysfunction of calcium-permeable channels was revealed as a possible inducer for cancer development, including gastrointestinal tumors [70]. Ca^2+^ is a main factor in the process of apoptosis and BCL-2 is blocking the programmed cell death induced by Ca^2+^-ionophores in various cell lines such as thymocytes, T-cell leukemia lines, and PC12 cells [71].

Other studies have shown that, in addition to the decreased activity of BCL-2, capsaicin produces an increase of cleaved caspase [33]. Caspase-3 is a protease that is systematically activated in the process of apoptosis [72]. Apoptosis may be achieved in a caspase-3 dependent or independent manner. The activation pathway of this protease is dependent of mitochondrial cytochrome c release and caspase-9 [73,74], and, by increasing cleaved caspase-3, capsaicin promotes the apoptosis of cancer cells in a multifaceted way: cell reduction or cell shrinking, blebbing of cytoplasmic membranes chromatin condensation and DNA disintegration. BCL-2 is known as the “antiapoptotic protein”, and a study from 2005 has revealed that the capsaicin intake promotes a reduction in the expression of BCL-2 [32]. As mentioned above, studies have shown that BCL-2 is responsible for the regulation of the transmembrane calcium fluxes [71]. In the same regard of antitumor effect of capsaicin in gastric cancer, it was revealed that capsaicin has the ability to inhibit the mutagenicity of different agents, such as aflatoxin B1 and the tobacco-specific nitrosamine4-(methylnitrosamino)-1-(3-pyridyl)-butanone (NNK)—two very potent carcinogens, by inhibiting their activation: [75] aflatoxin B1 is found in peanuts and different grains and shows mutagenic and teratogenic proprieties [76] while NNK has been linked with damage to the mitochondrial genome [77].

A recent study by Wang et al. showed a new epigenetic action of capsaicin capable of regulating cell growth in gastric cancer [28]. The authors showed that capsaicin promotes the activity of histone acetyltransferase hMOF inducing a positive feedback loop ending in cell cycle arrest and apoptosis in SGC-7901 and MGC-803 gastric cancer cells. The antitumoral effects of capsaicin on gastric cancer seem to be dose-dependent, as a Meta-Analysis demonstrated that a low intake exhibits protective effects against gastric cancer [78,79].

#### 2.2.2. Carcinogenic Effects of Capsaicin in Gastric Cancer

One of the carcinogenic factors involved in the development of gastric cancer is *Helicobacter pylori* [80]. The link between capsaicin and *Helicobacter pylori* in the development of cancer has been studied and results did not confirm interactions between the two counterparts connected to the risk of developing gastric cancer [58]. As already mentioned, p53 is an important factor in cancer development, and *Helicobacter pylori* may cause the inactivation of the P53 gene in gastric cancer through different mechanisms such as mutations or deletions [81], while capsaicin may counterbalance this negative effect of *Helicobacter pylori*. However, a high intake of capsaicin has proven to increase the risk of diffuse-type gastric cancer, and the mechanism has not yet been expounded [58]. It is known that intestinal-type gastric cancer is distinguished by overexpression of HER2 while diffuse-type gastric cancers are characterized by amplification of c-met receptor and aberrations in the EGFR kinase pathway [82]. Capsaicin’s connection with the EGFR pathway has been the subject of many studies, but its dose-dependent carcinogenic effect has not been definitively demonstrated. Furthermore, in vivo studies on mice have revealed capsaicin’s effect of promoting gastrointestinal tumor development in mice [83]. Other studies regarding gastric cancer have suggested a cocarcinogenic effect of capsaicin in some murine models (i.e., N-methyl-N’-nitro-N-nitrosoguanidine-induced gastric cancer) but not in other carcinogenesis models, such as the one induced by azoxymethane [84,85]. A large case-control study demonstrated the potential of capsaicin to stimulate the development of cancer cells in consumers of spicy food [86]. However, human populational studies measuring the dietary intake of capsaicin may suffer from significant limitations and bias related to nondifferential measurement errors, recall bias, selection and sampling bias, flaws in statistical methods, and the presence of various confounding variables [56,86]. As such, improving the design of these studies and analyzing larger data sets may yield more relevant results. Overall, even though prooncogenic effects have been cited in a variety of papers, the relevance of these studies is uncertain and further research is needed to confirm these results.

### 2.3. Colorectal Carcinoma

Colorectal cancer is the third most commonly diagnosed cancer in males and the second in females [87]. The majority of colorectal cancers (around 90%) are adenocarcinomas [88]. Other cancer types are less common: neuroendocrine, squamous cell, adenosquamous, spindle cell, and undifferentiated carcinomas [88]. Overall, 5–10% of all patients are affected by hereditary colorectal cancer syndrome. Lynch syndrome and familial adenomatous polyposis are two of the most common syndromes among colorectal cancer patients [89]. The incidence of colon cancer and daily diet are strongly linked [90]. However, while some studies have suggested a possible role of capsaicin-rich diet in carcinogenic processes of colonic mucosa [91], other numerous studies have indicated an anticancer effect of capsaicin [92,93,94]. One possible explanation of the anticancer properties of capsaicin in colorectal cancer is associated with Cyclin D1 degradation and 20S proteasome activity [42]. Cyclin D1 is a subtype of cyclin regulating cell cycle advancement from phase G1 to phase S [95]. Cyclin D1 expression is high in colorectal cancer due to the abnormal adenomatous polyposis coli or β-catenin genes [96]. The ubiquitin–proteasome is a regulatory complex with important roles in managing cancer development and consists of many factors that include E3 ubiquitin ligases, ubiquitin hydrolases, ubiquitin, and ubiquitin-like molecules [97,98,99]. Cyclin D1 is associated with ubiquitin and then relocated to the 26S proteasome, playing a main role in colorectal cancer by inducing the transition through the restriction point in the G1 phase [100,101].

#### 2.3.1. Anticarcinogenic Effects of Capsaicin in Colorectal Carcinoma

TRPV1 is expressed in the intestinal epithelial cells where a close connection with the epidermal growth factor receptor (EGFR) pathways has been emphasized [102]. Epidermal growth factor (EGF) is a 53-amino acid peptide that is responsible for cellular development, growth, survival, movement, programmed death, propagation, and differentiation [103]. The stimulation of EGFR is associated with an intrinsic activation of TRPV1. Further, TRPV1 initiates direct negative feedback on the EGFR, and blocking EGFR may keep cancer cells from developing and growing. Conversely, hyperactivation of EGFR pathways is stimulated by the lack of TRPV1 signaling which may promote cell proliferation increasing the risk of intestinal epithelium malignancies [104].

TRPV1 is a strong nonselective calcium channel that could powerfully affect the evolution of colorectal cancer cells [105], knowing from several studies that the imbalance of calcium influx is a stimulus for colon cancer development [105,106]. In an interesting study by Vinuesa et al., it has been revealed that mice with TRPV1 deficiency may develop cancer of the distal colon [107]. Calcium signaling is an important modulator in different cell cycle processes and numerous studies have highlighted its role in a variety of cellular mechanisms, involved in all major stages of cancer development [108,109]. For example, calcium signaling impacts both promotion and invasiveness, being involved in the regulation of cell proliferation and apoptosis through the Ca^2+^/calmodulin complex [110].

Another possible link is the effect of capsaicin on P53, a suppressor gene located on chromosome 17 [111,112] with an important role in cell cycle control and apoptosis. P53 may suffer various mutations and can contribute to the development of tumors [113]. The p53 mutant gene may have a pro-oncogenic function [114]. It has been shown that oncologic patients with mutations in the p53 gene may develop resistance to chemotherapy [115]. In a study performed in 2019, it was shown that treatment with TRPV1 agonist capsaicin can activate the p53 gene leading to inhibition of the development of colorectal cancer cells and stimulation of their apoptosis [105]. This confirmed previous similar findings by other authors [43,44,45].

The optimal dose of capsaicin needed to exhibit the maximum antitumoral effect in colon adenocarcinoma was investigated in a 2020 study by Nisari et al. [116]. The research team used silver staining of nucleolus organizer regions (AgNOR) in Caco-2 cells to determine the correspondent dose of capsaicin in the highest values of AgNOR and Total AgNOR area/nuclear area. This led to the conclusion that 50 to 75 uµ log concentrations of capsaicin solution is the most reliable dose for exhibiting the maximum chemopreventive effect.

**Figure 1:**
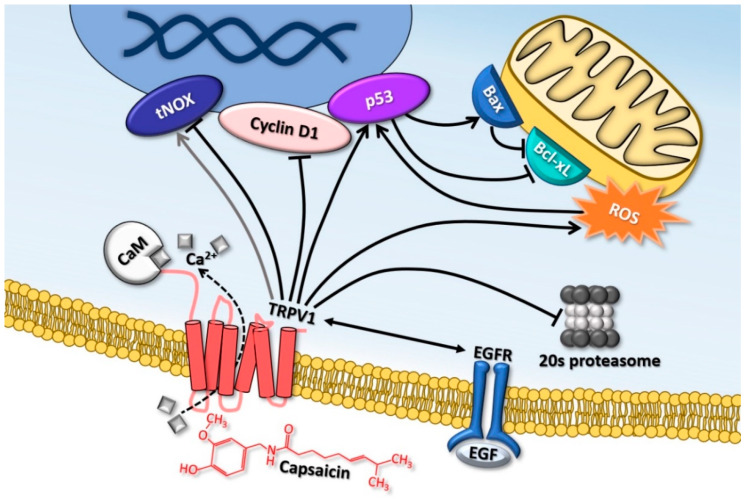
Major mechanisms mediating the antitumoral effects of capsaicin in colorectal carcinoma. The stimulating (arrowhead) and inhibiting (transverse bar) signaling effects of capsaicin are represented. Capsaicin activates the p53 gene causing increased protein levels, also favored by stimulating reactive oxygen species (ROS) production. P53 regulates the levels of Bax and Bcl-xL inducing apoptosis. The dual dose-dependent effect on tumor-associated NADH oxidase (tNOX) is also represented, which is upregulated at low capsaicin concentrations and inhibited at higher concentrations. Capsaicin causes a degrading of Cyclin D1 and decreased activity of the 20s proteasome. The inter-activation and signaling feedbacks of TRPV1 and EGFR receptors are represented with a double arrow. CaM = calmodulin.

Time-dependent antitumor effects of capsaicin on colon cancer cells were also described in a combined in vivo and in vitro study on Colo 205 cells and xenograft mice. The study showed that ROS generation and Bax/Bcl-xL modulation are of great importance in the induction of apoptosis in colon cancer cells [117], and these findings confirm other similar results [44,94], reinforcing the potential use of capsaicin as an anticancer agent in this pathology (Figure 1).

#### 2.3.2. Carcinogenic Effects of Capsaicin in Colorectal Carcinoma

As stated before, carcinogenic effects of capsaicin on colorectal carcinoma were also mentioned. An in vitro study by Liu et al. showed that capsaicin in low concentrations (between 0.1 and 10 µM) for 24 h can induce tumor cell growth and migration in HCT116 cells by upregulating the expression of tumor-associated NADH oxidase (tNOX). Furthermore, tNOX knockdown in HCT116 cells inhibits cell migration and proliferation suggesting this mechanism may mediate the oncogenic effect of capsaicin [60]. Conversely, a study published in 2020 deemed capsaicin as a safe food product in high doses as it failed to impact carcinogenesis progression in a preneoplastic colon cancer model in rats [118]. This data advises that further research is needed not only to establish the reliability of using capsaicin in colon cancer but also to find a safe dose for clinical use.

### 2.4. Cholangiocarcinoma

Cholangiocarcinoma (CCA) is a malignant tumor of the bile ducts, yielding high mortality, with intra- or extrahepatic locations, a factor related to the prognosis [119]. Research has demonstrated that CCA is resistant to a series of chemotherapeutic regimens including Fluorouracil (5FU), an analog of the pyrimidine uracil acting as a pyrimidine antagonist [120]. Fluorouracil interferes with DNA replication, RNA processing, and protein production [121,122] and inhibits cell proliferation [123].

In vivo and in vitro studies have shown that capsaicin is an important antiproliferative factor in various cancers and dietary use is now considered for its chemopreventive effect [55]. Among the antitumoral mechanisms cited in various studies, capsaicin has the capacity to diminish the activation of the hedgehog pathway, a signaling cascade with major roles in different mechanisms such as embryonic development and tissue homeostasis [124]. In CCA, capsaicin has yet again demonstrated its anticancer potential in an in vitro study which revealed that capsaicin can block the Hedgehog pathway activation and promote antitumor functions [34].

Capsaicin was proven effective in preventing CCA metastasis in a recent in vitro study on HuCCT1 cells by suppressing the expression of MMP-9 via the activation of the AMPK-NF-κB pathway [125]. The capsaicin-induced phosphorylation of AMPK inhibited the translocation and deacetylation of NF-κB p65 causing significant inhibitory effects on CCA cells migration.

Another interesting study has revealed promising results regarding the utility of capsaicin in the treatment of CCA. The authors employed in vitro and in vivo methods to show that capsaicin yields great results in association with 5FU by increasing the 5FU anti-tumor effects through 5FU-induced autophagy inhibition. Therefore, by using capsaicin in a complementary manner in the treatment of CCA, the chemoresistance met in CCA may be overcome [46].

Unfortunately, there is a scarcity of papers investigating the effects of capsaicin in CCA leading to grand anticipation of further studies, especially considering the poor prognosis and high mortality of this type of cancer and the paucity of therapeutic options in advanced disease.

### 2.5. Hepatocellular Carcinoma

Hepatocellular carcinoma (HCC) is the most common primary liver cancer, and its development is influenced by multiple factors that induce the uncontrolled division and transformation into cancer cells. The appearance of mutations in p53, PIK3CA, and β-catenin are common findings in the development of HCC [126]. Wnt/β-catenin is commonly altered in HCC and holds an important role in the differentiation and development of cancer [127]. The P53 mutant gene may have a pro-oncogenic function, p53 being a main cell-cycle catalyst protein that can suffer mutations and promote cancer development not only in the hepatic tissue but in different areas as well.

There is strong evidence regarding the expression of TRP channels in the liver, TRPV1 being involved in hepatocytes migration [128]. Moreover, the altered expression of TRPV1 holds a potential role in the development and progression of hepatocellular carcinoma. Thus, it is not surprising that in various research regarding capsaicin’s antitumor action, TRPV1 is the main catalyst [129]. However, other studies showing that capsaicin reduces HCC aggressiveness by inducing apoptosis in hepatic cancer cells have emphasized that this is a cumulative effect of different mechanisms that act against cancer cells: increase of intracellular Ca^2+^ production, the elevation of ROS, and regulation of the Bcl-2 protein [35].

The variety of actions that capsaicin exhibits in HCC may be organized into effects on tumor differentiation, genomic stability, cellular proliferation, oxidative stress, and angiogenesis [7]. Several studies have shown that capsaicin can induce cell cycle arrest and apoptosis in HepG2 cells mainly through the interaction with p53 and the AMPK pathway [35,48]. Moreover, capsaicin triggered apoptosis in HCC cells through other mechanisms such as ROS generation [50], endoplasmic reticulum stress [35], and phospholipase C-mediated Ca^2+^ release [49], and tumor necrosis factor-related apoptosis-inducing ligand pathway [51].

The suppression of angiogenesis may be promoted by capsaicin via the suppression of the vascular endothelial growth factor pathway and receptor. The use of monoclonal antibodies against VEGF has shown that HCC cells growth may be inhibited [130], and capsaicin is known to exhibit VEGF-induced cell proliferation [131], however, further studies are needed to confirm the antiangiogenic role of capsaicin in HCC.

Oxidative stress seems to play a major role in the metabolism of HCC cells [132] and capsaicin is able to modulate ROS causing apoptosis through NADPH-mediated pathways [133,134]. ROS generation can also lead to the accumulation of ceramide in some cancers [135], which may alter the cellular metabolism and trigger apoptosis via TRAIL activation [136]; however, these findings have not yet been corroborated in HCC.

#### 2.5.1. Synergistic Antitumoral Effects of Capsaicin and Sorafenib in Hepatocellular Carcinoma

An important area of research regards the combined effects of capsaicin and sorafenib, a kinase inhibitor considered to be the main treatment course for HCC in the advanced disease stage. In HCC, sorafenib significantly extended the median progression-free survival as compared with placebo [137], but as a relatively new molecule, it needs new ways to have its effects enhanced. A study from 2018 has shown that adding capsaicin to sorafenib has increased its apoptotic effect in HCC [47]. The study has displayed that the tumor activity and lifespan were diminished by the sorafenib and capsaicin compared to sorafenib or capsaicin alone. The ability to induce apoptosis and to diminish the tumor cell viability is dose-dependent, as a high concentration promotes a stronger effect. The same study has shown the increment of tumor sensitivity by an intensification of p-ERK and reduction of p-STAT3 signaling. P-ERK is a protein that opposes the MEK/ERK signaling cascade. The study has shown that the P-ERK levels are different in HCC patients. P-ERK is a crucial element of the RAf/MEK/ERK pathway. The Ras/Raf/MEK/ERK signaling pathway is involved in various cellular mechanisms that promote cancer cell development and is a key component in the development of HCC [138,139]. Sorafenib plays a major role in the treatment of HCC by inhibiting the Ras/Raf/MEK/ERK signaling cascade [140]. Signal transducer and activator of transcription–3 (STAT3) is a kinase that plays an important role in the induction, development, and dissemination of HCC [141]. A higher expression of STAT3 is linked with a worse prognosis. Sorafenib has the ability to inhibit tumor development by reducing STAT3 phosphorylation [142,143]. Therefore, combining sorafenib and capsaicin yields a synergic antitumor effect by inducing apoptosis and diminish tumor cell proliferation.

A necessary step in the spread of malignant HCC cells is the epithelial-mesenchymal transition, a complex process where MMPs play a major role, in particular MMP-2 and MMP-9 [144]. In a recent study, Dai et al. showed that the combined sorafenib and capsaicin treatment inhibits the development and metastasis of HCC cells, both in vitro and in vivo, through a variety of mechanisms including the downregulation of EGFR and PI3K/Akt/mTOR pathways and decreasing MMP-2, MMP-9, Bcl-2, and mesenchymal markers vimentin and N-cadherin [52].

#### 2.5.2. Carcinogenic Effects of Capsaicin in Hepatocellular Carcinoma

Even though numerous studies have shown that capsaicin has anti-inflammatory effects, promotes apoptosis, and reduces tumor cell proliferation, some research has pointed towards the mutagenic potential of capsaicin. Various studies have shown that capsaicin may be a prooncogenic substance that promotes cancer development, through different mechanisms [56,145]. In an early study published in 1952, researchers have demonstrated that rats fed with 10% chili peppers may be at risk of developing liver tumors [61]. This study was later criticized for not considering the potential effects of other hepatocarcinogens present in the rats’ diet [146]. The overall relevance of these controversial data is yet to be established.

### 2.6. Pancreatic Cancer

Globally, pancreatic cancer is the seventh cause of death caused by neoplasms [147]. This disorder is split into two categories of pancreatic cancer: pancreatic adenocarcinoma (85%, with a very poor prognosis) and pancreatic neuroendocrine tumors [148,149].

Ample studies have been developed investigating the possibility of improving survival in pancreatic cancer with new therapies [150]. As in other neoplasms presented above, capsaicin has been shown to have beneficial effects in pancreatic cancer. In a 2008 study, capsaicin has displayed apoptotic action against pancreatic cancer cells [53]. This action is mainly mediated by the activation of the mitochondrial death pathway which can be activated by ROS generation and Jun kinases (JNKs) activation [151]. Numerous stimuli can affect the mitochondrial membrane promoting the permeabilization of the outer membrane. Bcl2 is an antiapoptotic protein and an important factor in the regulation of membrane permeabilization. In addition to regulating the transmembrane calcium fluxes [152], Bcl-2 is blocking the programmed cell death induced by Ca^2+^-ionophores in different cell lines. Capsaicin intake was proven effective in reducing the expression of Bcl-2, offering another possible explanation for its antitumor effects in pancreatic cancer [32].

Other studies cited preventive effects of capsaicin against pancreatitis and it was revealed that chronic pancreatitis is an important risk factor for the development of pancreatic cancer [153]. Chronic pancreatitis favors the development of pancreatic cancer through the prolonged inflammatory process that leads to the production of increased concentrations of ROS and nitrogen species. Long-lasting oxidative stress is one of the main factors in the development of cancers via the induction of anomalies in cellular metabolism [154]. In an experimental mice model, a specific diet containing capsaicin over a period of eight weeks decreased the severity of chronic pancreatitis and significantly inhibited the progression of pancreatic intraepithelial neoplasia [54]. This was achieved most likely by inhibiting the inflammatory process within the pancreatic tissue as well as blocking the activation of the mutant Kras/ERK pathway.

The potential use of capsaicin in the treatment of pancreatic cancer is of particular interest due to the very low survival rates in these patients, despite the employment of multimodal therapy [155]. Therefore, the use of capsaicin alongside conventional chemotherapeutic drugs may be beneficial due to their combined and complementary effects.

## 3. Conclusions

In summary, capsaicin displays antitumoral effects in different stages of GI cancers through numerous molecular mechanisms. As mentioned above, the approach to therapies for these highly aggressive cancers is an evolving mission; new therapies and novel compounds are tested and perfected to increase survivability and quality of life. In multiple in vivo studies, capsaicin has shown anticancer activity by diminishing the progression of cancer cells on murine models by acting on various signaling pathways and cancer-related genes in HCC, CCA, pancreatic, and colorectal carcinoma. Recent studies have shown the potential of capsaicin in the treatment of HCC and CCA and its effects in enhancing the anticancer activity of other chemotherapeutic drugs employed in these patients. 

However, the existence of studies showing the carcinogenic effect of capsaicin in animal models of esophageal, gastric, colorectal carcinoma, or HCC should also be mentioned. These effects seem to be dose-dependent, which adds uncertainty regarding the chemopreventive effects of dietary usage due to intake dose and frequency inconsistencies. Some authors have observed in vitro carcinogenic effects of capsaicin, using doses in the range of daily intake, but in order to obtain cell proliferation, a prolonged exposure to the compound was required. This finding is balanced by the fact that capsaicin is quickly absorbed and then metabolized, so these effects might not actually be possible in normal dietary conditions. However, there is data to support that even capsaicin metabolites may carry on some effects which adds new layers of difficulty in correctly assessing the net effects and building adequate study designs. Further meta-analyses and human trials should clearly delineate the intricate effects of capsaicin, but the increasing number of publications and experimental models developed for evaluating the capsaicin efficiency in GI cancers demonstrate great interest in its potential and emerging evidence is encouraging.

## Figures and Tables

**Table 1:** The antitumoral effects of capsaicin on various gastrointestinal cancers in in vitro and in vivo models.

Cancer Type	Dose or Concentration/Duration of Application/Ingestion	Effect/Mechanism	Experimental Model	References
Esophageal squamous cell carcinoma	60 µM for 24 h	Glycolysis inhibition hexokinase-2 expression downregulation	in vitro (Het-1A, 293T, KYSE150, KYSE410, and KYSE510)	[40]
	50 µM for 24 h	inhibition of MMP-9 via AMPK activation	in vitro (Eca109)	[41]
Gastric cancer	10–300 µM for 12 h	Apoptosis, inhibition of cell proliferation, growth of cleaved caspase-3, decrease of the BCL-2	in vitro (Human gastric carcinoma AGS cells)	[33]
	10–200 µM for 24 h	Induction of apoptosis via a Bcl-2 mediated pathway	in vitro (Human gastric carcinoma AGS cells)	[32]
Colorectal carcinoma	20 mg/kg orally for 28 days	Limitation of the growth of polyps	in vivo (APC^Min+/^ mice)	[42]
	100 µM for 24 h	Suppression of the caspase-like action of proteasome 20S	in vitro (SW480, HCT116, LoVo, and Caco-2)	[42]
	40–160 µM for 24 h	Apoptosis via increasing expression of p53 and Bax	in vitro (HCT116 and HT-29)	[43,44,45]
Cholangiocarcinoma	150–200 µM for 24–96 h	Apoptosis, Modulation of Hedgehog pathway	in vitro (TFK-1 and SZ-1)	[34]
	150 mg/kg/day	decrease in tumor volume through increased apoptosis	in vivo (mice with tumor xenografts)	[46]
	40 µM for 24 h	Facilitates 5FU antitumor actions by activating the AKT/mTOR pathway	in vitro (QBC939, SK-ChA-1, and MZ-ChA-1)	[46]
Hepatocellular carcinoma	100 µM for 72 h	Apoptosis, p-ERK intensification, p-STAT3 reduction	in vivo (nude mice with PLC/PRF/5 xenograft)	[47]
	50–200 µM for 24 h	Induction of apoptosis via p53 and AMPK-mediated cell cycle arrest, PLC-dependent Ca^2+^ release, ROS modulation, and TRAIL induction	in vitro (HepG2 cells)	[35,48,49,50,51]
	80–120 µM for 24–72 h	Prevention of metastasis by inhibition of EMT and the EGFR and PI3K/Akt/mTOR pathways (combined with sorafenib)	in vitro (LM3)	[52]
	5 mg/kg intraperitoneal injection for 28 days	Inhibition of tumor growth, proliferation, invasion, metastasis by activation of autophagy and apoptosis (combined with sorafenib)	in vivo (BALB/C nude mice with LM3 xenografts)	[52]
Pancreatic cancer	100–200 µM for 24 h	Apoptosis, activation of mitochondrial death pathway	in vitro (AsPC-1, BxPC-3, and PANC-1)	[53]
	2.5–5 mg/kg, 3–5 days a week, oral intake for 39 days	Inhibition of tumor growth	in vivo (mice xenograft)	[53]
	10–20 ppm Capsaicin supplemented diet	Inhibition of cell proliferation in preneoplasic lesions by blocking Hedgehog and Kras/ERK pathways	in vivo (Pdx1-Cre and LSL-Kras/G12D mice with chronic pancreatitis via caerulein injection)	[54]

**Table 2:** Carcinogenic effects of capsaicin on various gastrointestinal cancers.

Cancer Type	Dose	Effect	Experimental Model	References
Esophageal squamous cell carcinoma	15 µM	increased cell proliferation	in vitro (Eca109 cells)	[57]
Gastric cancer	90–250 mg/day oral intake	increased risk of carcinogenesis (especially diffuse type gastric cancer)	Human case-control study	[58]
	5g/kg/day oral intake	Cocarcinogenic	in vivo (Sprague–Dawley rats treated with MNNG ^1^)	[59]
Colorectal carcinoma	≤10 µM	increased cell migration and proliferation	in vitro (HCT116 human colon cancer cells)	[60]
Hepatocellular carcinoma	10% chili pepper oral intake	stimulating carcinogenesis	in vivo (rats)	[61]

^1^MNNG = N-methyl-N’-nitro-N-nitrosoguanidine.

## Data Availability

Data sharing not applicable.
